# Adaption and validation of the Rwandese version of the Young Mania Rating Scale to measure the severity of a manic or hypomanic episode

**DOI:** 10.1186/s12888-024-05890-1

**Published:** 2024-06-18

**Authors:** E. Musoni-Rwililiza, C. J. Arnbjerg, N. U. Rurangwa, M. G. Bendtsen, J. Carlsson, P. Kallestrup, E. Vindbjerg, D. Gishoma

**Affiliations:** 1 College of Medicine and Health Sciences, University of Rwanda, Kigali, Rwanda; 2 Center for Global Health, Department of Public Health, Aarhus University, Aarhus, Denmark; 3 Competence Centre for Transcultural Psychiatry (CTP), Mental Health Centre Ballerup, Ballerup, Denmark; 4 Department of Clinical Medicine, University of Copenhagen, Copenhagen, Denmark; 5 University Teaching Hospital of Kigali (CHUK), Kigali, Rwanda

**Keywords:** Bipolar disorder, Hypomania/mania, Young mania rating scale, Cross-culturally adaptation, Validation, Low resources settings

## Abstract

**Background:**

Bipolar Disorder is one of the most incapacitating diseases among young persons, leading to cognitive and functional impairment and raised mortality, particularly death by suicide. Managing a manic episode and developing new and more effective treatment modalities requires sensitive and reliable instruments. This study aims to translate the English version of the YMRS questionnaire into Kinyarwanda, adapt it to the Rwandan context, and assess its validity.

**Methods:**

The original English version of The Young Mania Rating Scale questionnaire was translated into Kinyarwanda. The translation process followed a standardized approach, including back-translation, cross-cultural adaptation, and final adjustments. A total of 130 inpatients with bipolar disorder in a manic episode from CARAES Ndera Teaching Hospital were included. The descriptive statistics and test–retest correlations were carried out, as well as the CFA for validation and Rasch-analysis.

**Results:**

The Rwandese version of The Young mania rating scale had an adequate internal consistency (Cronbach’s alpha = 0.90). Item 11 provided the lowest standardized loading in both ratings (0.51 and 0.55). The second lowest loading involved the highly correlated item pairs 5 & 9, with item 5 loading 0.51 in rating 1 and item 9 loading 0.57 in rating 2. The remaining loadings ranged from 0.59 to 0.79. This relatively narrow range indicated that a fit to a Rasch model was plausible if excluding item 11.

**Conclusion:**

The findings demonstrate that the translated YMRS, the R-YMRS, can be used as a reliable and valid instrument for assessing mania in the Rwandese population in clinical and research settings. However, the results supported using an unweighted total score of 32 and removing items 5, 9, and 11. Studies on this revised scale with an added interview guide for less-trained clinical staff are recommended.

## Background

Over the past 50 years, several assessment tools for mania have been developed and evaluated [1–8]; Secunda et al. 1988. This work is essential as Bipolar Disorder (BD) is one of the most incapacitating diseases among young persons, leading to cognitive and functional impairment and raised mortality, particularly death by suicide [9]*.* BD is strongly associated with lower productivity levels, functional and social impairment, high prevalence of clinical and psychiatric comorbidities, and premature mortality [10]*. *Managing a manic episode and developing new and more effective treatment modalities requires sensitive and reliable instruments.

Eighty-five percent of the world's population resides in low- and middle-income countries (LMICs). Yet, the evidence regarding BD's psychopathology, management, and course is poorly described in these countries [11]. Estimates suggest that less than 10% of people with BD in LMICs get psychiatric care; [12–14]. Two recent systematic reviews find that there have been no intervention studies on the psychosocial treatment of BD from low-income countries and only 21 intervention studies from lower-middle-income countries on BD [15, 16].

There are several instruments to assess manic symptoms [17]. The Young Mania Rating Scale (YMRS) is one of the most widely used and oldest scales to measure manic symptoms. An obstacle to conducting research in many countries is the lack of validated assessment tools.

In Rwanda, a Sub-Saharan low-income country with 15 psychiatrists and 14 million people in 2023, no systematically validated instrument for assessing the severity of manic symptoms is available. Consequently, this study aims to translate the English version of The Young Mania Rating Scale (YMRS) questionnaire into Kinyarwanda, adapt it to the Rwandan context, and assess the validity of the Kinyarwanda version.

## Methods

### Study design

This study was conducted at the CARAES neuropsychiatric hospital Ndera, Kigali. The ethical committee at the hospital approved the study, the Ethical Committee at the University of Rwanda with the approval notice number 056/CMHS IRB/2021, and the Rwandan National Council of Sciences and Technology (NCST).

### Participants and data collection

All participants were recruited from the acute psychiatric unit of the Ndera neuropsychiatric hospital located 17 km from Kigali City, where they were hospitalized. CARAES-Ndera Hospital is a mission health facility, yet the government of Rwanda supports the hospital by providing human resources to the hospital and assisting in its management, and the singular neuropsychiatric hospital in the country with inpatient care that offers specialized healthcare in psychiatry and neurology.

The inclusion criteria were a diagnosis of BD type I or II that meets DSM-IV diagnostic criteria given by a trained psychiatrist, current manic or hypomanic state, and age ≥ 18 years. In contrast, the exclusion criteria included insufficient understanding of Kinyarwanda or deafness. Each patient signed an informed consent form. A senior psychiatrist examined each participant both to confirm the BD diagnosis and to evaluate the severity of the episode using the global clinical impression scale for BD [18]. Following the assessment by the senior psychiatrist, two raters simultaneously administered the YMRS questionnaire. The composition of each rating team varied, consisting of either a junior psychiatrist and a clinical psychologist or a mental health nurse and a clinical psychologist. All raters were trained in the administration of the YMRS by the investigator, who is also a psychiatrist.

### The instrument

The YMRS is a semi-structured interview rating scale with eleven items to estimate the severity of manic episodes [5]*.* The assessment includes the patients’ subjective reports and clinical observations made by the interviewer. Each item's objective is to assess the patient's level of symptom intensity, making the rating scale valuable for ongoing assessments of hypomanic or manic symptoms and the effects of the treatment in both academic and healthcare settings. The YMRS total score varies between zero and 60. The scale consists of 11 items, each rated on a 0–4 scale, except for items 5, 6, 8, and 9, assigned double weight, with higher scores indicating greater severity of manic symptoms. Young et al. [5], who developed the YMRS, originally proposed the weighting of these items. They found that these four items had the highest inter-rater reliability and were the most discriminating items between manic and non-manic states. Specifically, items 5, 6, and 8 assess the severity of grandiosity, increased activity or energy, and decreased need for sleep, respectively, core features of manic episodes in BD. Item 9 assesses the severity of the thought content associated with manic symptoms (i.e., racing thoughts). Subsequent studies have also supported the validity of the double-weighting of these items. For example, studies have shown that the double-weighted items have higher correlations with other measures of manic symptom severity, such as the Mania Rating Scale and the Clinical Global Impression-Severity Scale (CGI-S), compared to the other items on the YMRS [18, 19]. Furthermore, studies have shown that the double-weighted items are more sensitive to changes in manic symptom severity over time and can be used to assess treatment response in patients with BD [19, 20].

Overall, the double-weighting of items 5, 6, 8, and 9 on the YMRS appears to be a clinically valid approach to weighting the items on the scale based on their clinical significance and discriminative power.

The developers have demonstrated that the scale shows good psychometric properties. Interrater reliability of 0.93 for the total score and a high concurrent validity, such as a correlation between the YMRS total score and an independent global rating of 0.88. Due to its high reliability and validity coefficients, the YMRS is the one that is most frequently used in clinical trials [5]*.* As a result, the YMRS is commonly utilized as a reference for the concurrent validation of other instruments [7, 8, 21–25].

### Translation

The translation process followed a standardized approach for cross-cultural adaptation and validation [26]. Two bilingual translators whose mother tongue was Kinyarwanda produced two independent translations of the original English version of YMRS. They had different academic profiles, and the second translator was not knowledgeable about BD. Discrepancies between the two translations were resolved in a discussion between the translators. Next, the two translators synthesized the results of the translations. Afterward, two bilingual translators whose mother tongue was English, individually back-translated the Kinyarwanda version into English separately while being blinded to the original English version. The back translation and the original YMRS were compared to check the validity and similarities.

Finally, an expert meeting was held with all translators and eight clinicians. Item by item, all versions, and back translations were discussed to agree on an optimal pre-version for semantic and conceptual equivalence between the original English and the Kinyarwanda version. A consensus was reached on the items, and the final translation was reviewed and approved. Before assessing the measurement properties, we conducted a final pre-test of the instrument through in-depth interviews with 10 nurses at Kibagabaga District Hospital and 20 psychology students from the University of Rwanda who volunteered to test the translated instrument.

### Statistical analysis

The analysis commenced with establishing the most suitable measurement model for validating the YMRS. As responses to the YMRS are summed in a single total score, representing a single trait, it carries the implicit assumption of uni-dimensionality. Based on previous validation studies and based on the authors’ review of the YMRS, this was considered a plausible baseline assumption, obviating the need for an initial exploratory analysis. In fact, in the original publication of the YMRS, it is stated that "the YMRS is intended to be a uni-dimensional rating scale measuring the severity of manic symptoms.” They explain that the items were chosen based on their relevance to the clinical concept of mania and were evaluated for their ability to discriminate between mania and other psychiatric disorders [5]*.* In another study, Berket al*.* (2006) evaluated the factor structure of the YMRS using exploratory and confirmatory factor analysis. They found that a single-factor solution provided the best fit to the data, supporting the uni-dimensionality assumption of the scale (Berk et al. 2006)*.*

As items 5, 6, 8, and 9 are assigned double weight, these items are implicitly assumed to better discriminate levels of mania than the remaining items. This would call for validation with either a 2-parameter logistic (2-PL) item response theory (IRT) model or a confirmatory factor analysis (CFA) model.

Based on the considerations above, a uni-dimensional CFA model was chosen to evaluate the assumptions of uni-dimensionality and item loading patterns. We also assessed modification indices for indications of substantial residual correlations, as these violate the assumptions of the model and inflate the loadings of the involved items [27]. Combinations of positive and negative residual correlations will also indicate multidimensionality [28]*.*

As described in the Results section, the CFA supported further testing with a Rasch model Rash [29], which assumes uni-dimensionality and tau equivalence — the latter corresponding to equal factor loadings in CFA.

The Rasch analysis was carried out in steps of testing and model modifications. Each step involves an assessment of item fit and the assumptions of local independence and no differential item functioning (DIF). Breaches of the latter two assumptions were accommodated within the graphical log-linear Rasch model [30]. This allows particular items to have different difficulties for different subgroups and for particularly closely related items to correlate freely. Items were only excluded from the model when their indicated misfit could not be resolved by modeling accompanying indications of local dependence and DIF.

Three software packages were used for the analysis. The descriptive statistics and test–retest correlations were carried out with Stata [31]*,* the CFA was performed with Mplus [32], and DIGRAM [33] was used for the Rasch-analysis.

### Sample size

The sample size in our study was determined and estimated using Gorsuch’s rule (Gorsuch, 1983; Hatcher, 1994; Suhr, 2006), which requires a sample size of five times the number of questions assessed, resulting in a minimum of 55 participants. Usually, in factor analysis, a sample size of approximately 100 subjects is considered a minimum when conducting a validity study (Gudgeon et al. 1994), while others suggest a minimum of five for the subject-to-item ratio (Factor analysis, 2003). Therefore, we aimed for a sample size of 130 patients, with a subject-to-item ratio of approximately 12:1.

## Results

### Descriptive statistics

One hundred and thirty patients, 65 females and 65 males, aged 18 to 67, participated in the study. Most of them were enrolled in their first week of hospitalization. Just 30% had completed senior secondary school, and roughly, half of the study population was unemployed. The mean age was 31.9 (SD = 9.6) years, and the mean age of onset of BD was 21 (SD 7.2) years, while the mean of hospitalizations was 4.5 (SD 3.93) times.

Poverty level categories (Ubudehe) are social stratification programs depending on household income in Rwanda. These categories range from A to E, categories A and B comprise households that are self-reliant, while C and D indicate partial dependency on social protective schemes (https://rwandapedia.rw/hgs/ubudehe/poverty-level-categories; https://www.rssb.rw/scheme/cbhi-scheme). In this study, the majority was in social class C, D or E while none was in social class A. Table 1 summarizes the sociodemographic and clinical characteristics.

**Table 1:** A summary of the sociodemographic and clinical characteristics of the sample

	***n ***= 130
***Age, mean yrs (SD)***	31.9 (9.6)
***Female, n (%)***	65 (50)
**Education level, n (%)**	
Primary school	40 (30.8)
Junior Secondary School	30 (23.1)
Senior Secondary School	34 (26.2)
University Bachelor’s degree	12 (9.2)
University Master degree	2 (1.4)
Vocational studies	2 (1.4)
No education	10 (7.7)
**Poverty level (Ubudehe), n (%)**	
Class A	0 (0.0)
Class B	35 (26.9)
Class C	44 (33.9)
Class D	34 (26.2)
Class E	11 (8.5)
Unknown	6 (4.6)
**Employment status, n (%)**	
Working	38 (29.2)
Un-employed	61 (46.9)
Retired	1 (0.8)
Student	5 (3.9)
Others	25 (19.2)
**Literacy, n (%)**	121 (93.1)
**Mean age of onset*, yrs (SD)***	21 (7.2)
**Bipolar subtype, n (%)**	
Bipolar I	125 (96.2)
Bipolar II	5 (3.9)
**Illness duration, mean *yrs (SD)***	12.5 (8.3)
**Numbers of episodes, n (%)**	
1–5	92 (68.2)
6–10	15 (11.1)
More than 10	28 (20.7)
**Somatic Comorbidities, n (%)**	13 (10)
**Hospitalizations (number in a lifetime period)**	3 [1–17] (Mean:4.5 / SD:3.9)

Data are mean (S.D.), median [IQR], or percentage (n) unless otherwise stated

### Factor analysis

As theorized before the analysis, the results revealed substantial residual correlations between items 5 & 9 (rating 1: M.I. = 74.62, StdYX = 0.76; rating 2: M.I. = 41.42, StdYX = 0.62). For items 1 & 2, a much less substantial residual correlation was found in rating 1 (M.I. = 13.83, StdYX = 0.557) and none in rating 2. Allowing the involved items to correlate freely resulted in a satisfactory overall fit (rating 1: χ2[42] = 57.458, *p* = 0.056, CFI = 0.99, TLI = 0.99; rating 2: (χ2[43] = 60.062, *p* = 0.044; CFI = 0.99, TLI = 0.99) and no further indications of model misspecification.

While item 6 provided the highest loading of all items in rating 1, there were no other indications of items 5, 6, 8, and 9 providing superior loadings. Item 11 provided the lowest standardized loading in both ratings (0.51 and 0.55). The second lowest loading involved the highly correlated item pairs 5 & 9, with item 5 loading 0.51 in rating 1 and item 9 loading 0.57 in rating 2. The remaining loadings ranged from 0.59 to 0.79. This relatively narrow range was considered to indicate that a fit to a Rasch model was plausible if excluding item 11.

### Rasch analysis

The baseline Rasch model was rejected on several accounts, including the expected local dependence between items 5 and 9 and under the discrimination of item 11. As reverting to a graphical log-linear Rasch model (GLLRM) to account for local dependence and the differential item functioning (DIF) did not resolve the misfit of item 11, this item was subsequently removed. Items 5 and 9 were also involved in various indications of misfit during the model search, including positive and negative local dependence on other items, DIF, and item misfit. While the former could indicate multidimensionality, no consistent or meaningful sub dimensionality could be inferred. Item 9 was more easily endorsed by women, but modeling this did not resolve the item fit. Splitting the sample to explore this issue indicated that the item was only under-discriminated in the female sample, although only significantly so for rating 1. As no solution could be identified within the constraints of the GLLRM to retain items 5 and 9, both were excluded. Finally, a separate modification was carried out for each rating. For rating 1, the model was adapted to allow for a lower location of item 1 for women than men. For rating 2, item 4 was allowed different locations for each age group, mainly as respondents in their 30 s would most easily report sleep reduction, conditioned on the trait level. The resulting 8-item model, adjusting for gender-based DIF of item 1, displayed an overall fit (rating 1: CLR = 49.11 df = 46, *p* = 0.3495; rating 2: CLR = 49.45 df = 42 *p* = 0.2002) and no evidence of unaddressed local dependence or DIF.

### Reliability

The internal consistency of the modified scale was indicated by a Chronbach’s α = 0.83 for rating 1 and α = 0.86 for rating 2. The total-score inter-rater correlation was r = 0.90. Table 2 shows the interrater agreement for each item.

**Table 2:** Interrater agreement:

	**Variables**	**Obs**	**Mean**	**St. dev**	**Kappa**	**St. Err**
1	Elevated Mood	130	2.4	1.1	**0.3**	**0.04**
2	Increased Motor activity/Energy	130	2.5	1.0	**0.3**	**0.04**
3	Sexual Interest	130	1.6	1.3	**0.4**	**0.04**
4	Sleep	130	2.5	1.2	**0.4**	**0.04**
5	Irritability	130	1.3	1.1	**0.4**	**0.04**
6	Speech (Rate/Amount)	130	1.9	1.1	**0.3**	**0.04**
7	Language/Thought disorder	130	2.1	1.1	**0.3**	**0.04**
8	Content	130	1.6	0.9	**0.4**	**0.04**
9	Disruptive/Aggressive behavior	130	1.5	1.2	**0.3**	**0.04**
10	Appearance	130	1.0	0.9	**0.3**	**0.05**
11	Insight	130	0.9	1.2	**0.5**	**0.05**

## Discussion

This study aimed to evaluate the internal construct validity and the reliability of the Rwandese YMRS in a sample of 130 patients hospitalized for a manic episode. The results strongly support using an unweighted total score for eight of the eleven items. The remaining items supply less information in the full sample or a subset of the sample. The scale was found to provide good internal consistency and excellent inter-rater reliability.

Unlike other studies [5, 18–20, 34], the instructed double-weighting of the score of items 5, 6, 8, and 9 were not supported in this study. The original idea of Young et al., stating that irritability, speech (rate and amount), grandiosity and aggressive behavior (items 5, 6, 8 and 9 respectively), which are core features of manic episodes in BD; seem not to be the case in the Rwandese population. Young et al. [5], who developed the YMRS, originally proposed the weighting of these items. They found that these four items had the highest inter-rater reliability and were the most discriminating items between manic and non-manic states. Among our sample, all participants were on high doses of medication, mainly haloperidol or chlorpromazine, first generation antipsychotics, which could explain why grandiosity, increased energy, and a decreased need for sleep have been suppressed. Similarly, Licht et al. also observed comparable outcomes using the Mania Rating Scale. Their latent structure analysis of 100 manic in-patients indicated that the scale items measuring mood, self-esteem, sleep, and sexual interest were less effective in testing the severity of mania, largely due to the patients' treatment with zuclopenthixol [35].

Furthermore, in this study, item 9 was more easily endorsed for women but also provided a poor fit with the female subsample. As the assessment of this issue involved splitting the sample into gender-based halves, the result is less reliable, and we did not pursue solutions with entirely separate scales for women and men. In contrast to this finding (Disruptive-Aggressive Behavior), no previous research has found that women endorse this item more frequently than men do. Most studies that have examined gender differences in YMRS scores have not reported significant gender differences in item 9 specifically [36, 37]*.*

The issue of double-scoring in our sample is aggravated by two of the involved items displaying a clear content overlap between irritability and aggression. The top response category of item 5, “Hostile, uncooperative; interview impossible,” is difficult to clearly distinguish from the two top response categories of item 9, “Threatens interviewer; shouting; interview difficult” and “Assaultive; destructive; interview impossible.” As it stands, it is by far the feature with the most impact on the total score, and our results do not support this practice.

While irritability and aggression are often associated with manic episodes, it is unclear whether they should be considered pivotal features of the YMRS construct. There is some psychometric support for including irritability and aggression in the YMRS. For example, several studies have found that irritability and aggression are common symptoms of manic episodes and positively correlate with YMRS scores [38, 39]. Additionally, Miklowitz and Johnson (2007) found that irritability and aggression were among the most discriminative symptoms for diagnosing BD, suggesting that they may be key features of manic symptomatology. On the other hand, Van Metter et al. [40] found that irritability and aggression were more severe among youth with Cyclothymic Disorder than among youth with non-bipolar diagnoses, but did not differ across bipolar disorder subtypes.

Furthermore, other studies have found that irritability and aggression are not necessarily central to the YMRS construct. For example, a study by Lukasiewicz et al. [41] found that YMRS items related to energy, speech, thought content, and behavior were more strongly associated with overall YMRS scores than irritability and aggression items. Similarly, a study by safer et al. [42] found that YMRS items related to elevated mood, increased motor activity, and grandiosity were better predictors of overall symptom severity than irritability and aggression items.

### Practical Implications

The psychometric results of our study suggest that item number 11 on insight provides insufficent information to contribute to unweighted total scores of YMRS in the studied population and, as such, reduces the precision of the measure. Besides, lack of insight is not included in the diagnostic criteria for mania in the DSM-5 (APA, 2013).

Including unweighted scores for items, 5 and 9 implies both a risk of overrepresentation of aggression, as well as under discrimination and gender bias. More research should preferable be carried out in this population to establish whether and how aggression may be relevant and valid to include as a single item. Finally, our data do not support the double weighting of items 5 (irritability), 6 (speech), 8 (content), and 9 (disruptive aggressive behavior).

The resulting adjustment of the YMRS in Kinyarwanda (R-YMRS) contains 8 items, all contributing a score from 0 to 4. Apart from the improved construct validity, this will be shorter and easier to use by clinicians. Moreover, in a context such as Rwanda, where there is a limited resource on mental health personnel and psychoeducation or pharmacological therapies, this may be tremendously helpful. First, it will allow clinicians and researchers to quantify the severity of manic symptoms easily and track changes in symptoms over time. This can be helpful in determining treatment efficacy and making decisions about medication adjustments or other interventions in a timely and appropriate manner. The R-YMRS can also aid in the diagnosis of BD. Other practical implications of the R-YMRS include its ability to predict relapse and hospitalization in individuals with BD. A higher R-YMRS score has been associated with a greater risk of relapse and hospitalization, indicating that the scale can be helpful in identifying individuals who may require more intensive treatment or monitoring. However, in a resource-constrained setting, it would have been beneficial with exact definitions for the items assessed, which the YMRS does not have. Without operational definitions, each evaluator utilizes their prior experience with psychopathology as a reference point to define the assessed item, which reduces the inter-evaluator reliability [43]. Moreover, there is no structured process for collecting data with the YMRS. For individual evaluators and evaluators without considerable expertise in recognizing and conceptualizing psychopathology, structured interviews or interview guidelines may be especially helpful [44]*.* Considering these factors, it may be useful to develop a small interview guide for the R-YMRS.

Overall, the R-YMRS is a valuable tool in the assessment and management of BD, with a range of practical implications for clinicians, researchers, and patients in Rwandan healthcare settings.

### Limitations

Our study is limited by the inclusion of only inpatients, and we only administrated the scale over a period of three weeks and did not administer the scale longitudinally to assess its sensitivity to symptom changes over a longer period. Furthermore, it is important to acknowledge that the clinicians administering the YMRS were relatively new to the instrument, having received one day of training prior to the study. While efforts were made to ensure consistency and reliability in scoring, the limited training duration may have introduced variability in assessment practices. In resource-constrained settings like Rwanda, where access to comprehensive training programs may be limited, providing detailed rating guidelines for the YMRS and clear operational definitions could enhance the quality of data collection and minimize inter-rater variability.’’.

## Conclusion

The findings demonstrate that the translated YMRS, the R-YMRS, can be used as a reliable and valid instrument for the assessment of mania in the Rwandese population in clinical and research settings. Among potential uses are to aid diagnosis, assess the severity of mania, help decisions about medical adjustments, and monitor changes in mood symptoms over time in patients with BD in an appropriate and time-efficient manner. However, results provided support for the use of an unweighted total score for eight of the eleven items, removing items 5, 9 and 11 with a total score of 32. Further studies on this revised scale with an added interview guide for less trained clinical staff tested over a longer period and in a district hospital would be of value.

## Data Availability

The datasets used in this study are available upon request. Interested parties can obtain access to these datasets by contacting the corresponding author.
